# Improved Spread Spectrum Aloha Protocol and Beam-Hopping Approach for Return Channel in Satellite Internet of Things

**DOI:** 10.3390/s23042116

**Published:** 2023-02-13

**Authors:** Liang Gou, Dongming Bian, Baogui Dong, Yulei Nie

**Affiliations:** 1 Nanjing Smart Constellation Information Technology Co., Ltd., Nanjing 210007, China; 2 College of Communication Engineering, Army Engineering University of PLA, Nanjing 210007, China; 3 Changzhou Huawei Electronic Co., Ltd., Changzhou 213144, China; 4 College of Integrated Circuit Science and Engineering, Nanjing University of Posts and Telecommunications, Nanjing 210023, China

**Keywords:** spread spectrum aloha, random access, beam-hopping, satellite communications, return channel, beam scheduling, maximum-weighted clique

## Abstract

This paper examines potential performances of the Spread Spectrum-based random access technique and proposes an Improved Spread Spectrum Aloha (ISSA) protocol for the return channel in satellite Internet of Things (IoT) based on the beam-hopping technique. The key design driver and detailed solution of ISSA protocol are presented in this work and it is shown that the proposed protocol achieves high throughput and low collision probability. To match user/traffic distribution, delay requirement and channel condition with beam allocation better, a low-complexity heuristic beam scheduling algorithm and a more effective Maximum-Weighted Clique (MWC) algorithm have been proposed. The heuristic algorithm considers the user/traffic distribution, inter-beam interference, and fairness primarily. However, the MWC algorithm gives considerations not only on above factors, but also on delay requirement and channel condition (path loss and rain attenuation) to maximize system capacity. The beam angle and interference avoidance threshold are proposed to measure the inter-beam interference, and the link propagation loss and rain attenuation are considered meanwhile in the channel condition. In the MWC algorithm, we construct an auxiliary graph to find the maximum-weighted clique and derive the weighting approach to be applied in different application scenarios. The performance evaluation of our ISSA protocol compared with the SSA protocol is presented, which achieves a gain of 16.7%. The simulation of the ISSA protocol combined with round robin, heuristic, and MWC beam scheduling for the return link in beam-hopping satellite IoTs is also provided. The results indicate that the throughput in nonuniform user distribution is much lower than in the uniform case without the beam scheduling algorithm. Through the application of the scheduling algorithm, the throughput performance can approach the uniform distribution. Finally, the degree of user satisfaction with different scheduling approaches is presented, which validates the effectiveness of heuristic and MWC algorithms.

## 1. Introduction

There is a growing requirement of terminals for the fixed/mobile broadband satellite Internet and IoT (Internet of Things) market. One huge challenge is that efficient multiple access protocols must be able to cope with a large network size and huge amount of users. It is a real obstacle to future broadband satellite communication systems and satellite IoTs that are characterized by a tremendous amount of users and more dynamic bursty traffic, which require that the system is able to accommodate this characteristic and a large number of users can access the system favorably in a short time.

To deal with the access of a tremendous number of users simultaneously and randomly, it is essential to develop efficient Random Access (RA) protocols. The traditional RA protocol, such as Aloha and Slotted Aloha (SA) have been implemented in satellite communication system extensively [1]. However, the large collision probability results in long delay and low throughput, which limits its application in a network circumstance with high load. For this, an enhanced version of the SA scheme, called Diversity Slotted Aloha (DSA) is proposed in [2] and applied today in satellite Time Division Multiple Access (TDMA) systems with low efficiency. Recently, Contention Resolution Diversity Slotted Aloha (CRDSA), that is, an improvement of DSA has been introduced in [3] and its performance outperforms SA and DSA. In CRDSA, each user transmits a fixed number of packet replicas in independently and randomly selected slots. A generalized manner of CRDSA named Irregular Repetition Slotted Aloha (IRSA) has been given in [4], characterized as the number of replicas generated randomly from an optimized Probability Density Function (PDF). In 2015, the Coded Slotted Aloha (CSA) protocol has been proposed in [5], whose basic idea is transmitting encoded segments of data packets instead of replicas. Strict time synchronization desired in these schemes based on the slot is achieved hardly in satellite communication systems due to the long propagation path and moving satellite, which may result in large, diverse and variant delay. Furthermore, signal bursts especially in satellite IoTs will make the time synchronization more difficult.

The Spread Spectrum Aloha (SSA) scheme has been presented in [6,7]. This scheme combined Code Division Multiple Access (CDMA) and Forward Error Code (FEC) in a physical layer design to maximize the RA throughput while keeping a low Bit Error Rate (BER). An Enhanced-SSA (E-SSA) scheme has been proposed in [8] that employs Successive Interference Cancellation (SIC) to achieve further improvement on RA throughput. SSA and E-SSA are unslotted RA schemes in which the packets are transmitted asynchronously without any replicas and time coordination. The collision is solved by the favorable relativity of spread-spectrum codes (e.g., m-sequence and Gold sequence). Nevertheless, the SIC technique requires sufficient diversity in received power or Signal to Interference plus Noise Ratio (SINR) for all users to achieve separation from the mixed signal, and the complicated processing calls for powerful computation ability. Hence, a more intricate receiver configuration is essential, which handles difficultly in satellite communication for limited payload and power.

Compared with traditional multi-beam technology, beam-hopping is more promising for improving capacity in nonuniform user and traffic distributions in satellite communication systems [9,10]. The purpose for beam-hopping is to allocate resources flexibly to meet time-varying and nonuniform traffic demand. Beam-hopping is based on multi-beam reflector or phased array satellite antenna that enable the change of beam direction, size and shape flexibly and fleetly. A phased array antenna also has the ability of beam forming, enabling the beams to cover different shape and size according to the system’s demand. For these, satellite communication achieves tremendous advancements benefiting from beam-hopping. First of all, small point beams make the power of the satellite and terrestrial equipment concentrate on the communication direction, which achieves a gain up to tens of dBs. Benefiting from this gain, high-throughput satellite communication and terminal miniaturization based on beam-hopping is available and gradually become a prospective trend in future satellite communication systems. In addition, the beams point to different cells in different slots and this is a more flexible and dynamic resource allocation mode. According to the user or traffic distribution, a different number of slots is allocated to different cells in a beam-hopping cycle. In other words, cells hold more terminals or traffic will be allocated more slots. Adversely, cells holding a small number of terminals may be allocated few slots or even no slots. In addition, the slot allocation can be adjusted dynamically with the change of terminal or traffic distribution. Hence, higher system throughput and user satisfaction can be achieved. In recent years, several methods have been proposed to schedule the beams to match the user distribution and system demand, such as Deep Learning [11] and Multi-Objective optimization [12]. To alleviate co-frequency interference among beams, a dynamic clustering method has been proposed to balance traffic among clusters in [13]. While a joint beam-hopping and precoding algorithm has been provided to realize the intra-cluster interference suppression and achieve near-optimal transmission capacity in this article.

However, the above methods do not consider the long-term reward, time-varying traffic demand, and channel condition. Otherwise, the conventional algorithms such as genetic algorithm have a long convergence time so that they cannot meet the real-time scheduling. In [14], a dynamic beam pattern and bandwidth allocation scheme based on DRL has been presented to realize flexible scheduling of multidimensional resources, such as time, space, and frequency. They give a cooperative Multi-Agents Deep Reinforcement Learning (MADRL) framework to maximize the throughput and minimize the delay. The authors of [15] explored a new framework in which a DRL-Powered Genetic Algorithm (GA) was used to fully meet dynamic changes in multi-beam satellite communication scenarios. This method shows excellent performance in throughput and fairness with dynamic change of traffic and environment.

Unfortunately, the above approaches are so complicated that the satellite cannot implement them effectively due to its limited computation resource. In addition, these approaches have considered system throughput but no consideration on different condition or service requirements, such as fairness, delay, channel conditions. In reference [16], the authors investigated an optimal beam-hopping policy in different conditions and QoS (Quality of Service) demand, and give a multi-action selection method based on Double-Loop Learning (DLL) to fulfill several targets simultaneously. A maximal User Service Weight Gain (maxUSWG) resource allocation algorithm has been proposed in [17]. The authors defined a user service weight gain to measure the traffic demand and delay sensitivity of each cell, and allocated resources to cells with larger weight gain until the power is exhausted or the number of working beams reaches a certain threshold. The simulation results indicate that this approach can improve QoS and resource utilization effectively.

Reference [18] aimed at the inaccurate positioning problem caused by different signal quality between user and multiple LEO satellites to propose a Flexible Beam-Hopping Control Algorithm (FBHCA) and decompose this problem into three sub-problems. These sub-problems are solved by three approaches or techniques, respectively, and the numerical results indicate that these approaches can improve the positioning precision obviously.

In Reference [19], the authors exploited the time-space-domain flexibility and power-domain flexibility of Non-Orthogonal Multiple Access (NOMA), and jointly optimized a beam-hopping scheduling, slot assignment of users, and power allocation to satisfy the traffic demand as much as possible.

A mathematical model of the beam illumination problem has been given in [20]. This model employs an interference-based penalty function to avoid precoding and the beam illumination problem has been modeled as a Binary Quadratic Programming (BQP). The Semi-Definition Programming (SDP) approach, and Multiplier Penalty and Majorization-Minimization (MPMM) based method have been provided to search for the local optimum. Finally, a low-complexity greedy algorithm has been proposed to minimize the use of precoding.

In future satellite Internet and IoT systems, a massive amount of users will look for access to satellites randomly. Subsequently, the probability that a good deal of users get access to a satellite simultaneously increases significantly and this will deteriorate access capacity greatly for severe collision. In order to prevent this circumstance, a high-efficiency random access protocol that can hold more terminals is needed urgently. Furthermore, the conventional multi-beam satellite communication systems waste a lot of beam and slot resources under nonuniform user/traffic distribution for their wide beams or multiple point beams and provide almost the same coverage to all cells no matter the distribution of users or traffic. Hence, a novel beam coverage mode is needed to provide more fair access opportunity to all users. At the same time, the dynamic change of user and traffic distribution requires a dynamic and flexible beam coverage scheme. The motivation of this paper is finding a random access protocol, combined with effective beam-hopping scheduling to reduce the collision probability and improve the access capacity. A more important matter is finding an efficient method to match the beam-hopping with dynamic user/traffic distribution effectively and accurately.

In this paper, we review the random access protocols and beam-hopping satellite communication, and propose an Improved SSA (ISSA) protocol to improve system capacity. Then, two beam scheduling algorithms have been proposed to deal with the nonuniform and dynamic distribution of users/traffic. The contributions of this paper are detailed as follows.

- An ISSA random access protocol has been proposed to improve the access capacity. The preamble is introduced in each beam-hopping slot that is used to accommodate the head of users’ burst signal. The capacity analysis and numerical simulation are conducted for this protocol and the results indicate that performance of the ISSA protocol outperforms the conventional ones.
- Give the mathematical model and analysis of beam scheduling in beam-hopping satellite IoT systems. Firstly, the influencing factors have been analyzed, and a mathematical optimization model based on linear programming has been presented. Secondly, the performance analysis indicates the potential of beam-hopping in satellite IoT systems.
- Two novel beam scheduling algorithms have been presented. To deal with the nonuniform user/traffic distribution in beam-hopping satellite systems, a heuristic beam scheduling algorithm with low complexity is given, and Maximum-Weighted Clique (MWC) beam scheduling algorithm is proposed to improve the system capacity further. Simulation results indicate that the throughput is improved greatly.

The organization of this paper is presented as follows. Section 2 gives the system model and the ISSA protocol design. The mathematical model of beam scheduling and two scheduling algorithms have been proposed in Section 3. Then, the performance analysis of our proposed protocol and beam scheduling algorithm are presented in Section 4. The simulation results are given in Section 5. Section 6 presents some remarks about satellite communications. Finally, Section 7 gives the conclusion of this paper.

## 2. System Model and Protocol Design

A system model of general multiple access in beam-hopping satellite communication systems and ISSA protocol design is described in this section.

### 2.1. System Model

**Figure 1:**
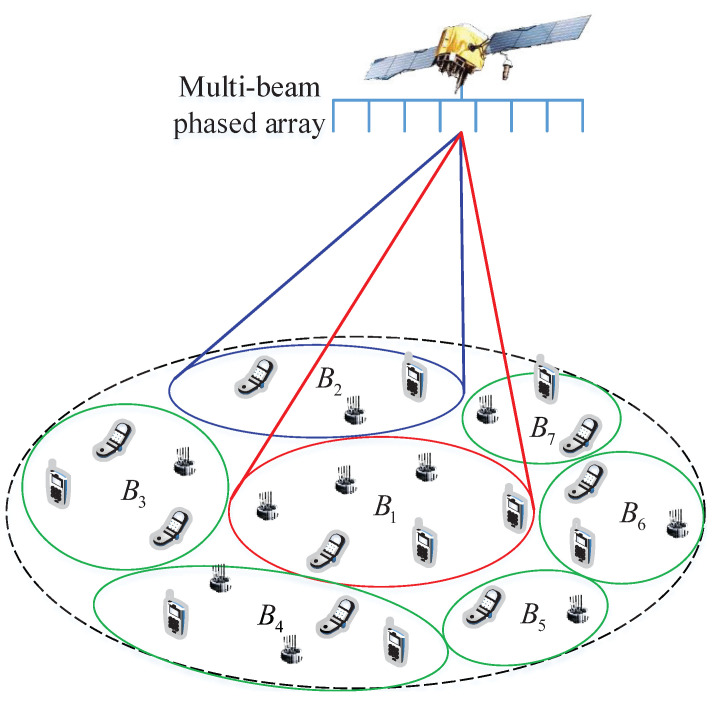
RA model in beam-hopping satellite system. $B_{1}∼B_{7}$ denote seven cells in this system.

The basic system model as shown in Figure 1 is illustrated as a satellite with multi-beam reflector antenna or phased array antenna providing IoT access service to the covered Satellite Terminals (STs). To describe the system model conveniently and exactly, several definition are given in the following.

Beam-hopping slot: The minimum time unit of slot allocation and beam hopping. On-board computer allocates each beam-hopping slot to cells and beams change their pointing at the beginning of each beam-hopping slot.

Beam-hopping cycle: The period of beam hopping contains several, dozens or hundreds of beam-hopping slots generally. The on-board computer generates a beam-hopping pattern based on the beam-hopping cycle.

**Figure 2:**
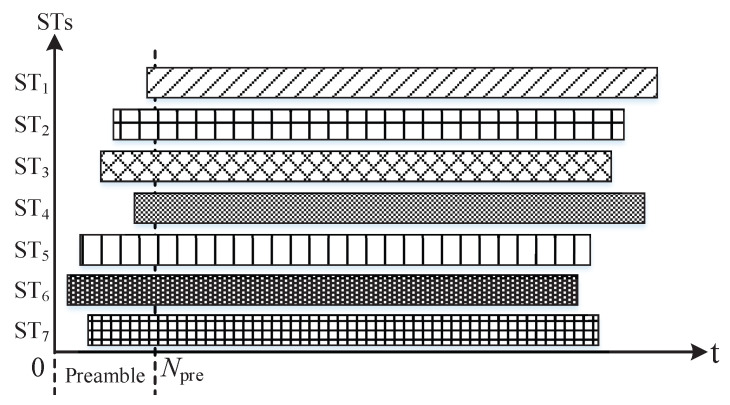
Demonstration of ISSA protocol.

Preamble: It is defined as the chips in the header of each beam-hopping slot as shown in Figure 2.

Several beams illuminate a subset of cells in one beam-hopping slot. The subset of cells illuminated in a beam-hopping slot is determined by the beam-hopping pattern, which gives the solution by which the beam illuminates which cell in which slot. In this model, there may be one or several beams that can be used in the same beam-hopping slot. In a beam-hopping cycle, a cell may be illuminated once or many times, or not illuminated even, which is also determined by the beam-hopping pattern. In the preamble time of a beam-hopping slot, STs covered by beams with traffic already arrived try to access the satellite.

### 2.2. Protocol Design

To ensure the delay performance of user traffic, an ISSA protocol is designed to deal with the access of STs to satellite as soon as possible, i.e., STs will transmit a signal to the satellite once the beam arrives.

The number of chips in the preamble may range from dozens to thousands, which is determined by the network scale and performance requirement. A long preamble time makes the system hold more STs in one beam-hopping slot, whereas more time (or slot) resource is wasted. In an optimized system, a trade-off should be achieved between the throughput and resource consumption.

In our designed protocol, STs encoded their data frame using the predetermined Spread Spectrum (SS) codes, and choose the transmitting time dependent on the return link time synchronization algorithm to ensure that the signal received at the satellite is within the preamble time. A satellite will receive a mixed signal combined by all STs’ SS signal transmitted in the same beam-hopping slot. Benefiting from the favorable relativity of SS codes, one ST can decode its information correctly as long as no other STs’ signal falls into the same or neighboring chip slot and SINR is fulfilled.

**Figure 3:**
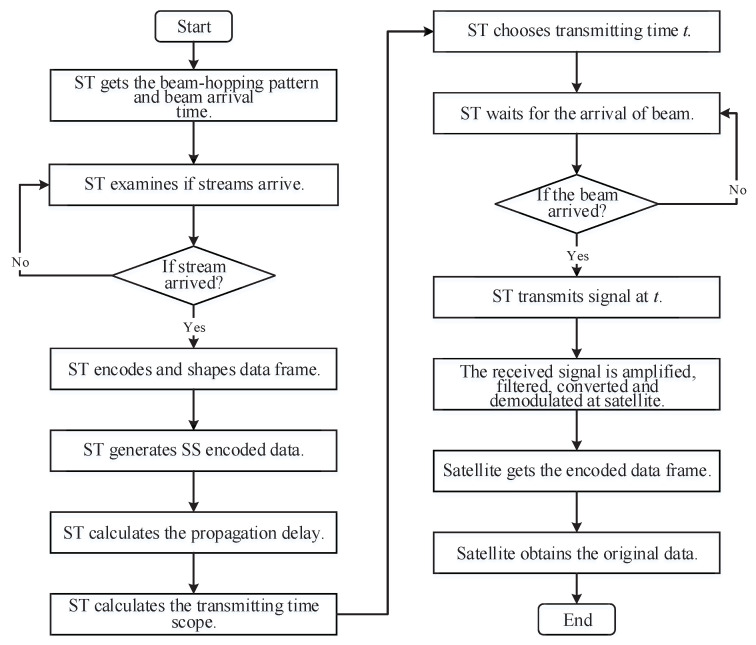
ISSA flowchart in beam-hopping satellite communications.

The flowchart of the ISSA protocol in beam-hopping satellite communication systems is shown in Figure 3. The whole access process of our protocol is shown as follows.

Step 1: ST gets the beam-hopping pattern and beam arrival time from satellite broadcast information.

Step 2: ST examines if traffic has arrived. If yes, go to the next step. Otherwise, repeat this step.

Step 3: ST encodes the arrived original traffic and forms an encoded data frame.

Step 4: ST generates SS encoded data from the original data frame with a predetermined PN sequence.

Step 5: ST calculates the propagation delay from it to the satellite using the position (or ephemeris) information of the satellite and its own position.

Step 6: ST calculates the transmitting time range $\left[t_{0},t_{0}+\Delta \right]$ using the beam arrival time, propagation delay, and preamble time.

Step 7: ST chooses the transmitting time *t* during $\left[t_{0},t_{0}+\Delta \right]$ randomly to make sure that the signal reception at the satellite is aligned with chip slots in the preamble.

Step 8: ST waits for the arrival of the beam. If it arrives, go to the next step. Otherwise, keep on waiting.

Step 9: ST transmits a signal at *t* after the modulation, frequency conversion, and amplification of SS-encoded data.

Step 10: After the signal arrives at the antenna of the satellite, the received signal is amplified, filtered, converted, and demodulated at the satellite.

Step 11: Satellite obtains the encoded data frame of STs through the de-spread-spectrum of the demodulated signal.

Step 12: The original data frame is obtained from de-framing and decoding at the satellite.

The above flowchart contains almost all steps from the generation to reconfiguration of all STs’ original data at the satellite. In this flowchart, the signal spectrum of all STs is expanded with a given spread spectrum factor. For one ST signal, the others are considered as interference or noise so that this system is self-interfering. Hence, more STs transmit signals in one beam-hopping slot implying larger interference. Once the number of STs transmitting simultaneously reaches a certain level, no one’s information can be recovered at the satellite. Compared with the SSA protocol, ISSA is designed specifically for beam-hopping satellite communication systems. In the ISSA protocol, using the differences in receiving time of STs that lay in the preamble of each beam-hopping slot, the satellite receiver is able to extract different STs’ signals even if they use the same spread spectrum code. Simultaneously, the ISSA protocol is also applicable to other multiple access scenarios, as long as the receiving time of each user’s signal to the receiver is controlled. The SSA protocol uses different spread spectrum codes to distinguish different users, and extracts signals of different users from the mixed signal.

## 3. Beam-Hopping Scheduling Algorithm

### 3.1. Considerations on Beam Scheduling

#### 3.1.1. Beam-Hopping Approaches

In beam-hopping satellite communication systems, the coverage area of a satellite is much larger than the terrestrial mobile base station, and different cells contain different number of STs due to the heterogeneity of ST distribution. Generally, three spectrum usage schemes have been used in beam-hopping satellite systems.

- Full scheme. All beams employ the full frequency band allocated to the satellite, and the beams working in the same slot have to be apart sufficiently from each other to avoid or diminish the co-channel interference.
- Reuse scheme. This scheme divides the whole frequency band into several mutually disjoint sub-bands so that different beams are able to use different sub-bands to illuminate adjacent cells simultaneously.
- Hybrid scheme. This scheme is a hybrid of the full and reuse schemes. A beam can employ full or sub-bands, and the beams working simultaneously cannot employ the same frequency, even only partially overlap, unless there is sufficient spatial isolation among them.

#### 3.1.2. Signal Model

In multi-beam and beam-hopping satellite communication systems, the spectrum has been reused among different beams to improve spectrum efficiency. However, co-channel interference is a vital problem for spectrum reuse and sufficient isolation must be established between beams with spectral overlap. When the beam j(j∈J) illuminates the cell k(k∈K), the satellite return link gain matrix $H_{i}=\left[h_{k,j}|j\in J,k\in K\right]$ from the ST’s transmitter to the on-board receiver at slot *i* is defined as

(1) $$H_{i}=A\cdot G_{t}\cdot G_{r}$$

where $A=diag\{\alpha_{1},\alpha_{2},\cdots ,\alpha_{K}\}$ gives the channel gain matrix, $G_{t}=\left[g_{k,j}^{t}|j\in J,k\in K\right]$ and $G_{r}=diag\left\{g_{1}^{r},g_{2}^{r},\cdots ,g_{J}^{r}\right\}$ denote the transmitting and receiving antenna gain matrices, respectively, from cell STs to satellite. $h_{k,j}$ is the channel gain from STs in cell *k* to satellite using beam *j*. The beam occupancy condition at slot *i* is represented by $X_{i}=\left[x_{k,j}|x_{k,j}=0,1\right]$, and $x_{k,j}=1$ indicates that *k*-th cell is illuminated by beam *j*; otherwise, $x_{k,j}=0$. In order to reduce the complexity, $H_{i}\left(k,j\right)=0$ if $x_{k,j}=0$ is set.

In beam-hopping satellite communication systems, high spectrum utilization achieved through full spectrum reuse results in co-channel interference. When one beam illuminates one cell, the signal to interference plus noise (SINR) is expressed as Equation (2):

(2) $$\Gamma_{i,k,j}=\frac{h_{k,j}\cdot P_{k,j}}{N_{0}\cdot B+\sum_{k^{\prime }\in K,k^{\prime }\neq k,x_{k^{\prime },j}=1}h_{k^{\prime },j}\cdot P_{k^{\prime },j}}$$

where $p_{k,j}\in P_{i}$, $P_{i}$ is the transmitting power matrix at slot *i* and $p_{k,j}$ is the total transmitting power in cell *k* using beam *j*, $P_{k,j}$. *B* is the beam bandwidth, and $N_{0}$ is the noise power spectral density.

#### 3.1.3. Interference Avoiding

In [21], a multi-beam reflector antenna system has been designed to combine beam-hopping and size reduction of effectively used spots, which enables the higher-cell-reuse scheme of 12 reduced to 4. This paper demonstrates that implementation of beam-hopping in this case only requires a switching matrix, and if the dual-polarization feeds are used, it is possible to reduce the number of antennas for the reuse scheme from 4 to 2.

At present, the phased array antenna is preferable in beam-hopping satellite communication systems. The phased array antenna changes the shape of the pattern by controlling the feed phase of the radiation unit in the array antenna. Controlling the phase can change the direction of the maximum value of the antenna pattern to achieve the purpose of beam scanning. Compared with the traditional multi-beam antenna, the phased array antenna has great advantages. The main feature is that it can freely control the beam and can flexibly change the direction, width, and shape of the skip beam [22]. The phased array antenna uses electronic methods to achieve non-inertial beam scanning, so it is also called an electronic scanning array. Its beam direction is controllable, the scanning is flexible, and the antenna gain is higher.

The phased array antenna can use a fixed gain attenuator to change the size of the beam in the hardware structure, that is, each array element is connected to a fixed gain attenuator to form a window function sequence. Firstly, the width and center angle of the beam are determined. The linear frequency modulation sequence is used as the weighting vector, and the beam width and center angle are adjusted by changing the parameters of the sequence. The beam coverage can also be controlled by increasing or decreasing the sub-array, and the phase of the weighting vector can be designed to control the beam pointing [23]. The gain of the phased array antenna [24] is shown as follows,

(3) $$G_{T}\left(\theta \right)=G_{max}{\left(\frac{J_{1}\left(u\left(\theta \right)\right)}{u(\theta )}+36\frac{J_{3}\left(u\left(\theta \right)\right)}{{\left(u\left(\theta \right)\right)}^{3}}\right)}^{2}$$

where θ is the off-axis angle, $J_{1}$ and $J_{3}$ represent the first- and third-order Bessel function, respectively, and the peak gain $G_{max}$ is

(4) $$G_{max}=\frac{\eta^{\prime }N^{2}\pi^{2}}{{\theta_{3dB}}^{2}}$$

where $\eta^{\prime }$ is the antenna efficiency, usually 0.6∼0.7, *N* is the number of phased array elements, $\theta_{3dB}$ refers to the lobe width of the antenna, and 

(5) $$u\left(\theta \right)=2.07123\frac{sin\theta }{sin\theta_{3dB}}$$

**Figure 4:**
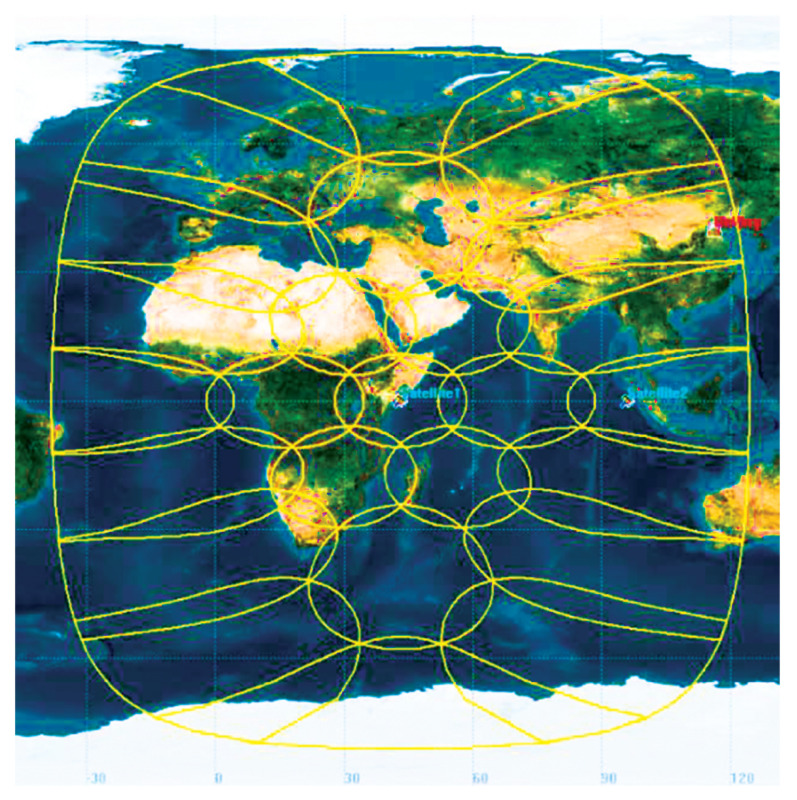
An example of cells in a GEO satellite communication system.

Under the condition of limited system resources, phased array beam-hopping technology can well solve the communication problem of small capacity and relatively dispersed geographical location. The different beams generated by the on-board multi-beam phased array payload in the broadband satellite communication access system will cover different regions. Further, the number of users and the traffic demand of users in different regions have spatiotemporal differences. The phased array antenna can control the pointing and shaping of the beam through the agility characteristics, and disperse the services within the multi-beam coverage as evenly as possible into different beams. It can give full play to the advantages of a controllable beam number, variable beam pointing, and beam shaping of the on-board multi-beam phased array payload, and improve the resource utilization of the broadband satellite system. Figure 4 gives an example of cell distribution in a GEO satellite communication system.

### 3.2. Optimization of Beam Scheduling

If all cells are assigned slots equally, the cells containing a large number of STs may be incapable to fulfill all STs’ access request. Whereas, the cells containing a few or no active STs may waste the beam and slot resources. In other words, the beams and slots are not tuned with the ST and traffic distribution. The beam-hopping is proposed to resolve this problem. In beam-hopping satellite communication systems, the number of assigned slots to cells in a beam-hopping cycle can be adjusted according to the distribution of STs or traffic. The more STs or traffic in a cell, the more slots are assigned to it in a beam-hopping cycle.

In the current beam-hopping cycle, the on-board processing module will calculate and determinate the beam and slot allocation for the next beam-hopping cycle based on the ST distribution, access request, and traffic characteristics in all cells. The scheduling of beams and slots can be formulated as an optimization of maximum system capacity as follows:

(6) $$\begin{array}{cc} \max & \sum_{i}\sum_{j}\sum_{k}\left\{x_{i,j,k}\cdot D_{k}\cdot R_{i,k}\right\} \end{array}$$

(7) $$\begin{array}{cc} s.t.\; & \frac{\sum_{i}\sum_{j}x_{i,j,k}}{I\times J}\leq P\left(k\right) \end{array}$$

(8) $$\begin{array}{cc} & \sum_{i}\sum_{k}x_{i,j,k}\leq \;J \end{array}$$

(9) $$\begin{array}{cc} & \theta_{i,k_{m}k_{n}}\geq \theta_{c},\;\forall i\in I,\forall k_{m},k_{n}\in K \end{array}$$

(10) $$\begin{array}{cc} & \sum_{i}\sum_{j}x_{i,j,k}\geq \tau^{\prime } \end{array}$$

where $x_{i,j,k}$ is the illuminated state at slot *i*, $x_{i,j,k}=1$ indicates beam *j* illuminates cell *k* at slot *i*; otherwise, $x_{i,j,k}=1$. $D_{k}$ is the number of terminals in cell *k*. $P_{k}$ is the proportion of STs in cell *k* to the number of STs in the system. $\theta_{c}$ stands for inference avoidance threshold between any two beams’ pointing that is denoted by an angle and $\tau^{\prime }$ is the minimum number of slots that is allocated to a cell in a beam-hopping cycle. Constraint (7) gives the fairness constraint, which is used to ensure the cells that hold more STs can be allocated more slots. *J* is the number of beams, and hence, (8) is used to maintain practically available satellite beams. Constraint (9) denotes that the pointing between any two beams is larger than threshold $\theta_{c}$, where $\theta_{i,k_{m},k_{n}}$ is the pointing angle between beams that illuminate cell $k_{m}$ and $k_{n}$. The latitude, longitude, and height of satellite and cell center can be transformed into XYZ coordinates in the Earth-Centered Earth-Fixed (ECEF) coordinate system. The beam pointing is the line from satellite to the center of the cell. In the ECEF coordinate system, we can calculate the linear equation of the beam pointing through the XYZ coordinates of satellite and the cell center. Then, the angle between two beam pointings will be calculated. Constraint (10) is the minimum number of slot constraints that are used to ensure STs access the satellite in a beam-hopping cycle. Different circumstances ask for different settings. For instance, in signaling interaction, the beams need to illuminate each cell at least once per set time cycle.

### 3.3. Heuristic Scheduling Algorithm

The above optimization scheduling is an NP-Hard problem. In this section, a heuristic beam scheduling algorithm is presented in consideration of several factors:

- Distribution of STs or traffic. The cells cover more STs or traffic will be allocated more slots.
- Inter-beam interference. The angle between any two beams’ pointing with frequency overlap must be larger than the beam width or a setting angle.
- Fairness of STs. The slot allocation for all cells is proportional to the number of STs or traffic contained in.
- Delay requirement. Beam-hopping satellite communication system provides Real-Time (RT) service (call, video call, etc.) and non-RT service (data, picture, video, etc.). RT service is sensitive to delay, which is an important criterion. Generally speaking, the target of scheduling strategy is maximizing the throughput for NRT data and minimizing the delay for RT data.
- Channel condition. The coverage area of a satellite is large and different cells may characterized as different channel condition for diverse path losses and weather conditions. Better channel conditions bring higher transmission capacity. The cell where the footprint is located has the largest channel capacity under the same condition due to the minimum path loss.

The detailed depiction of the beam scheduling algorithm is presented in Algorithm 1 to maximize the access capacity in satellite IoTs. It is a heuristic algorithm based on the greed method. In this algorithm, a cell is selected randomly from all cells at each iteration and will be examined if the allocated slots to it is over the proportion of its users/traffic in the system. If yes, this cell will not be allocated a new slot in this beam-hopping cycle; or else, it will be allocated. Then, the interference among beams is another criterion to determine whether to allocate this beam and this slot to the cell. Here, the interference threshold $\theta_{c}$ is used to make this decision. If the angle of beam pointing between any two selected cells is less than $\theta_{c}$, while with frequency overlap, these two cells will not be allocated beams in the same slot. The above two criteria examine the fairness and interference limitation, respectively.
**Algorithm 1** Heuristic Beam Scheduling Algorithm**Require**:
T, I, J, K.**Ensure**:
H.1:**Initialize** Let H=Ø, $H={\left\{0\right\}}_{1\times K}$.2:Calculate the proportion of the ST number in all cells using T, and store them in P (the total number of slots is I×J).3:**for** slot *i* in I **do**4:   Let $K^{\prime }=K$.5:   **for** beam *j* in J **do**6:     Let a=17:     **while** (a==1) **do**8:        Choose a cell *k* from $K^{\prime }$ randomly.9:   **if** the allocated slot proportion for cell *k* is smaller than P(k) **then**10:         **if** the beam that points to cell *k* is not interfered with other beams **then**11:              Append {i,j,k} to H.12:              Let T(k)=T(k)+1.13:              Let $K^{\prime }\left(k\right)=0$ or delete *k* from $K^{\prime }$.14:            Update the traffic size or required number of slots for cell *k* according to allocated slot and traffic arrival of cell *k*.15:              Let a=0.16:          **else**17:              Let $K^{\prime }\left(k\right)=0$ or delete *k* from $K^{\prime }$.18:          **end if**19:        **else**20:          Let $K^{\prime }\left(k\right)=0$ or delete *k* from $K^{\prime }$.21:        **end if**22:     **end while**23:   **end for**24:**end for**

Otherwise, additional factors or demands may be considered in the beam scheduling. For example, the beam-hopping is applied in the management and control system, whose satellites need broadcasting signaling information to users through the forward channel. In this way, the forward beams should cover all cells in a fixed time cycle. Likewise, the return beams should do so to receive the users’ signaling messages, such as register, entry, leave, resource request, and so on.

### 3.4. Maximum-Weighted Clique Scheduling Algorithm

In the current beam-hopping cycle, the on-board processing module generates several sets for the beam scheduling in the next beam-hopping cycle and calculates the beam-hopping pattern based on these sets.

V: the cell/vertex set, contains the cells which need access to the satellite to transmit signaling or service data in the next cycle, denoted as $V=\{v_{k}\}$ (k∈K).

W: the weight set is the set of weights for all cells/vertices, denoted as $W=\{w_{k}\}$ (k∈K), which is determined by ST or traffic distribution, delay requirement, and channel condition of cell *k*. The more STs/traffic in cell *k*, $w_{k}$ will be larger. Likewise, the cells with RT service and good channel condition will be assigned large weights. There is a certain corresponding relation between $w_{k}$ and $v_{k}$. Only if $v_{k}$ is in V, the corresponding value of $w_{k}$ is positive. Or else, $v_{k}=0$.

Θ: the set of angles among beams is the set of angles between any two beams’ pointing (assume these two beams illuminate cells $k_{m}$ and $k_{n}$, respectively), denoted as $\Theta =\{\theta_{k_{m},k_{n}}\}$ ($k_{m}\in K$, $k_{n}\in K$ and $k_{m}\neq k_{n}$).

**Figure 5:**
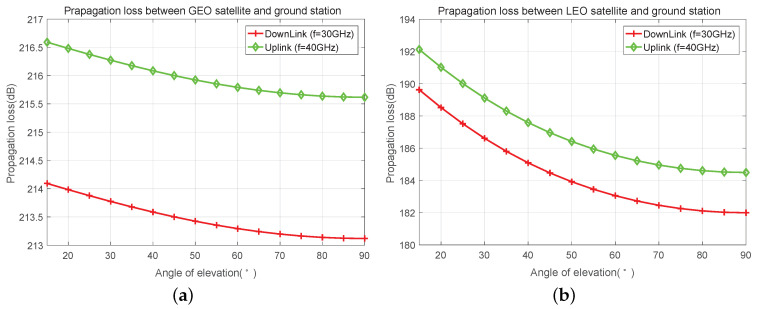
Propagation loss between satellite and ground station with different angle of elevation in the Ka frequency band. (**a**) Propagation loss between GEO satellite and ground station. (**b**) Propagation loss between LEO satellite with 1000 km height and ground station.

In Figure 5, we give the free-space propagation loss against angle of elevation, which is represented as $\theta_{e}$ in GEO and LEO satellite communication systems with ka frequency band. It is shown that the propagation loss increases along with the reduction of $\theta_{e}$, especially in GEO satellite communication systems, the gap of propagation loss between maximum and minimal $\theta_{e}$ is up to about 8 dB.

**Figure 6:**
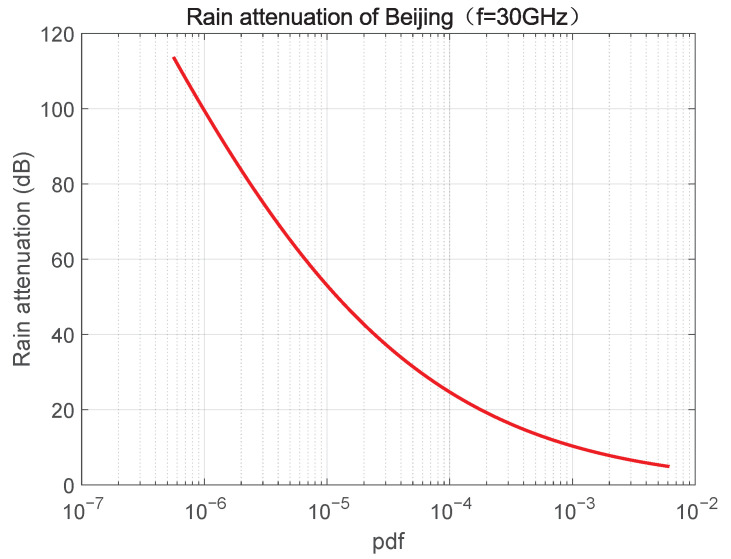
Rain attenuation of Beijing with f=30 GHz.

We calculate rain attenuation in Beijing with f=30 GHz as shown in Figure 6 according to [25]. A very wide range of rain attenuation from a few dB to over 100 dB is presented in this figure. In other words, the weather changes have a great influence on link equality. Table 1 shows the rain attenuation in Beijing, Shanghai, Sanya, and Chongqing with 99.9% and 99.99% availability.

In the satellite communication system, the coverage area of a satellite is very large. Generally speaking, a satellite can cover a radius of several hundreds kilometers to tens of thousands of kilometers. In such a large coverage area, the weather conditions in different cells may vary greatly, resulting in significant diversity in link equality between satellite and users in different cells. Link quality will affect the link capacity and Quality of Service (QoS). The propagation loss between GEO/LEO satellite and terrestrial users is presented in Figure 5a and Figure 5b, respectively. In addition, rain attenuation in Beijing is also shown in Figure 6. It can be seen that different elevation angles have different propagation losses, especially in LEO communication systems. Similarly, it is shown in Figure 6 that the change of rain attenuation is very large, and the rainfall directly affects the magnitude of rain attenuation. If the weather conditions of cells are very different, then the diversity of link states will be large correspondingly. Therefore, it is necessary to take propagation loss and rain attenuation into consideration in beam scheduling through a novel approach. It is a natural choice to improve the throughput and QoS of the whole system by giving priority or larger weight to the cells with low path loss and rain attenuation. To achieve this, we propose the maximum-weighted clique algorithm with full scheme.

Before designing the maximum-weighted clique, we first introduce an auxiliary graph in which each vertex is assigned a weight. We then show that the beam scheduling problem can be transformed into finding a maximum-weighted clique problem in the auxiliary graph.

We can construct an auxiliary graph G(V,E) where $V=\left\{v_{k}\right\}\left(k\in K\right)$, which is defined above. If cell $k_{m}$ and cell $k_{n}$ are not adjacent and $\theta_{k_{m},k_{n}}>\theta_{c}$, cell $k_{m}$ and cell $k_{n}$ can be illuminated by two different beams in the same beam-hopping slot. In the other words, the edge connecting $v_{k_{m}}$ and $v_{k_{n}}$ must fulfill the following two conditions:

- Cells $k_{m}$ and $k_{n}$ are not adjacent.
- $\theta_{k_{m},k_{n}}>\theta_{c}$.

In addition, we use a link/edge $e_{\left\{k_{m},k_{n}\right\}}\in E$ between $v_{k_{m}}$ and $v_{k_{n}}$ to denote this case.

For a clique $Q=\{v_{k_{1}},v_{k_{2}},\cdots ,v_{k_{q}}\}$ in the graph, any two vertices have been connected by an edge. In addition, the weight of Q is the sum of all vertices’ weight in this clique:

(11) $$W_{Q}=\sum_{v_{k}\in Q}w_{k}$$

Maximum-weighted clique (MWC) is the clique with the largest weight sum of all vertices, and is denoted as $Q_{m}$. Assuming Q is the clique set based on the auxiliary graph G(V,E),

(12) $$Q_{m}=\max_{Q\in Q}W_{Q}$$

is equivalent to the expected maximum number of users/STs or expected maximum size of traffic or expected maximum gain of the system. In this paper, the maximum gain is used as the measure standard of our beam scheduling. This gain is the scheduled cells’ or vertices’ weight sum, involves not only throughput but also quality of service. Hence, rational weighting of each vertex is the key problem. As presented above in this paper, distribution of STs or traffic, delay requirement, and channel condition should be considered in the weighting of vertices. The fairness of STs and inter-beam interference will be also considered in the scheduling algorithm.

(13) $$w_{k}=\alpha_{k,1}\times \beta_{k,1}+\alpha_{k,2}\times \beta_{k,2}+\alpha_{k,3}\times {\left(log_{2}\left(1+10^{\frac{\beta_{k,3}}{10}}\right)\right)}^{-1}$$

where $\alpha_{k,1}$, $\alpha_{k,2}$ and $\alpha_{k,3}$ are the weights of cell *k* used to represent the importance of user/traffic distribution, delay requirements, and channel conditions. $\beta_{k,1}$ is the proportion of user/traffic in cell *k* to the totality in the system, $\beta_{k,2}$ is the service priority related to delay requirements, and $\beta_{k,3}$ denotes the comprehensive effect of path loss and rain attenuation.

**Figure 7:**
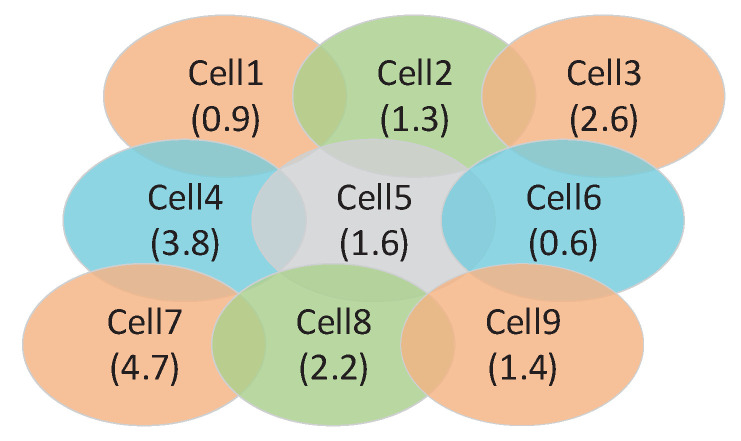
A satellite coverage with 9 cells.

An example of a beam-hopping satellite communication system with nine cells is presented in Figure 7. Assume that two cells can be illuminated simultaneously when they are not adjacent. That is to say, the angle between two beams that illuminate two nonadjacent cells is larger than $\theta_{c}$. In this way, cell 1 can be illuminated simultaneously with three other ones (cell 3, cell 7, and cell 9). However, cell 5 cannot be illuminated at the same time with any other cells.

**Figure 8:**
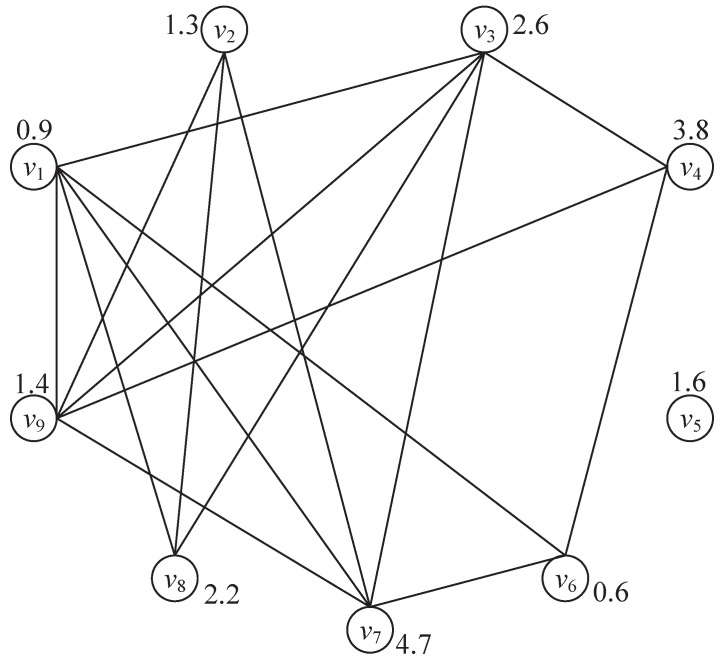
Graph model.

After constructing the graph model and assigning weights to each vertex in the graph as presented in Figure 8, the beam scheduling problem is then transformed into finding the maximum-weighted clique in such a graph. As shown in Figure 8, several cliques can be found, such as $\left\{v_{1},v_{3},v_{7},v_{9}\right\}$, $\left\{v_{1},v_{6},v_{7}\right\}$, $\left\{v_{1},v_{3},v_{8}\right\}$, $\left\{v_{2},v_{7},v_{9}\right\}$, $\left\{v_{5}\right\}$ etc., in which $\left\{v_{1},v_{3},v_{7},v_{9}\right\}$ is the clique with the maximum weight 0.9+2.6+4.7+1.4=9.6.

Assume that the total number of vertices in G(V,E) is *K*. First, arrange all vertices in descending order of their weight. For example, given in Figure 8, vertices in G will be arranged into V=$\{v_{1}\left(v_{7}\right)$, $v_{2}\left(v_{4}\right)$, $v_{3}\left(v_{3}\right)$, $v_{4}\left(v_{8}\right)$, $v_{5}\left(v_{5}\right)$, $v_{6}\left(v_{9}\right)$, $v_{7}\left(v_{2}\right)$, $v_{8}\left(v_{1}\right)$, $v_{9}\left(v_{6}\right)\}$. Without loss of generality, we denote all vertices with $V=\{v_{1},v_{2},\ldots ,v_{K}\}$ where $w_{1}\geq w_{2}\geq \ldots \geq w_{K}$ and $N(v_{k})$ is the adjacent vertex set of $v_{k}$ that contains all its adjacent vertices.

Let $Q_{k}$ be the clique with the largest weight ($Q_{m}$) in the graph, which only contains vertices of $S_{k}=\left\{v_{k},v_{k+1},\ldots ,v_{K}\right\}$ and let $W_{Q_{k}}$ be the weight of clique $Q_{k}$. The algorithm starts with k=K and iteratively considers more vertices until all vertices in G are considered, and stops with $Q_{1}$ is found.

From Algorithm 2, we can see that there are two cases when vertex $v_{k}$ is searched. If $Q_{k+1}\cup \left\{v_{k}\right\}$ is also a clique, then $Q_{k}=Q_{k+1}\cup \left\{v_{k}\right\}$ and $W_{Q_{k}}=W_{Q_{k+1}}+w_{k}$. Or else, we need to find out a new clique $Q_{k}$ in the subgraph that consists vertices $S_{k}=\left\{v_{k},v_{k+1},\ldots ,v_{K}\right\}$. Based on the descending ordering of vertices’ weights, the joining of clique vertices starts from the smallest *j* that $v_{j}\in S_{k}$, where $S_{k}$ is updated by $S_{k}=S_{k}\cap N\left(v_{k}\right)$ if $S_{k}\neq Ø$. The joining of clique vertices and the update of $S_{k}$ will continue until $S_{k}=Ø$ and the maximum-weighted clique including $v_{k}$ is found. Then, the temporary maximum-weighted clique will be picked up by comparing $W_{Q_{k+1}}$ and $W_{Q_{k}}$, and the larger one will be hold. The process will continue until $Q_{1}$ is achieved. The details of the maximum-weighted clique algorithm are given in Algorithm 2.
**Algorithm 2** Maximum-Weighted Clique Beam Scheduling Algorithm.**Require**:
G=(V,E), $W=\{w_{1},w_{2},\ldots ,w_{N}\}$.**Ensure**:
$Q_{m}$.1:**Initialize:**2:Let $Q_{k}$ = Ø, k∈{1,2,…,K−1};3:Let $Q_{K}=\left\{v_{K}\right\}$;4:Let $W_{Q_{k}}=0,k\in \left\{1,2,\ldots ,K-1\right\}$;5:Let $W_{Q_{K}}=w_{K}$;6:Let $Q_{m}=Q_{K}$;7:Let $max=W_{Q_{K}}$8:Let $S_{k}=\left\{v_{k},v_{k+1},\ldots ,v_{K}\right\},k\in \left\{1,2,\ldots ,K\right\}$;9:**Scheduling:**10:**for**k=K−1:1**do**11:   **if** $\left\{v_{k}\right\}\cup Q_{k+1}$ is also a clique **then**12:     $Q_{k}=Q_{k+1}\cup \left\{v_{k}\right\}$;13:     $W_{Q_{k}}=W_{Q_{k+1}}+w_{k}$;14:   **else**15:     $Q_{k}=\left\{v_{k}\right\}$;16:     $W_{Q_{k}}=w_{k}$;17:     $S_{k}=S_{k}\cap N\left(v_{k}\right)$18:     **if** $S_{k}$ = **then**19:        **if** $W_{Q_{k}}>max$ **then**20:          $Q_{m}=Q_{k}$;21:          $max=W_{Q_{k}}$;22:          mc=k;23:        **end if**24:        return;25:     **end if**26:     **while** $S_{k}\neq$ **do**27:        $j=min\{j|v_{j}\in S_{k}\}$;28:        $Q_{k}=Q_{k}\cup \left\{v_{j}\right\}$;29:        $W_{Q_{k}}=W_{Q_{k}}+w_{j}$;30:        $S_{k}=\left(S_{k}-\left\{v_{j}\right\}\right)\cap K\left(v_{j}\right)$;31:        **if** $S_{k}=$ **then**32:          **if** $W_{Q_{k}}>max$ **then**33:             $Q_{m}=Q_{k}$;34:             $max=W_{Q_{k}}$;35:             mc=k;36:          **end if**37:        **end if**38:     **end while**39:     **if** mc≠k **then**40:        $Q_{k}=Q_{k+1}$;41:        $W_{Q_{k}}=W_{Q_{k+1}}$;42:     **end if**43:   **end if**44:**end for**45:**if**$|Q_{m}|>J$ **then**46:   $Q_{m}=Q_{m}\left(1:J\right)$;47:**else**48:   return;49:**end if**

In addition, the number of vertices in the found maximum-weighted clique cannot exceed the number of beams in the beam-hopping satellite communication system. In other words, the cardinality of $Q_{m}$ cannot be larger than *J*. Hence, at the end of the Algorithm 2, the cardinality of the maximum-weighted clique needs to be judged. If the cardinality of it is greater than the number of beams, the first *J* elements in the maximum-weighted clique are extracted to form the terminal maximum-weighted clique.

The maximum-weighted clique algorithm (Algorithm 2) is used to schedule beams and cells in each beam-hopping slot, which could be used to replace lines 5∼23 of Algorithm 1 but keep the 9-th line to ensure fairness and lines 11∼14 for data storage and update. Otherwise, the weights of vertices should be updated after each scheduling of the beam-hopping cycle according to the remaining business volume, delay requirement, and link status.

## 4. Performance Analysis

In a SSA system, co-channel interference from other STs can be approximately equivalent to Gaussian noise even without any power control. We assume SNRs of all ST signals are identical and denoted as γ before they are SSed. Hence, the noise power $N_{0}$ can be denoted as follows:

(14) $$N_{0}=10^{-\frac{\gamma }{10}}$$

Assume that the number of STs in a cell whose signal arrived at the satellite in a beam-hopping slot is *T*, and the SS factor is η. Then, SINR for an ST is

(15) $$\gamma^{\prime }=-10\times \mathrm{lg}\left(N_{0}+\frac{(T-1)\times 2}{\eta }\right)$$

From $\gamma^{\prime }$, the modulation-and-encoding set and the required Bit Error Ratio (BER), $T_{max}$ can be achieved, which is the maximum number of STs that can be accessed simultaneously in one beam-hopping slot through the same beam.

In our analysis, we assume Additional Gaussian White Noise (AWGN) and no power unbalance. Assuming a Poisson process for traffic arrival, the number of STs in cell *k* that prepare to access is $T=\lambda \times \tau \times D_{k}$ when a beam arrives. In a beam-hopping slot, if $T\leq T_{max}$, the satellite may decode some STs’ signals. Otherwise, all STs’ signals cannot be recovered. In the first instance, if any two STs’ signal arrival times are within one chip time, the signals of these two STs will collide and no information can be decoded. To improve the recovery ability, we assume that an ST’s information can be recovered only if the time interval between its own arrival time and that of the others are equal to or larger than one chip. In such case, the probability $p_{0}$, which is the probability that a packet is decoded successfully, is denoted as [26]

(16) $$p_{0}=\sum_{t=1}^{T_{max}}\frac{T\cdot N_{pre}}{t!}e^{-T\cdot N_{pre}}$$

and the throughput expressed in packets per beam-hopping slot is

(17) $$S=T\cdot N_{pre}\cdot \sum_{t=1}^{T_{max}}\left(\frac{T\cdot N_{pre}}{t!}\cdot e^{-T\cdot N_{pre}}\right)$$

Obviously, the throughput is expressed in bits per second. It is obtained by multiplying *S* with the number of bits per packet, and divides the results by the time length of a beam-hopping slot.

## 5. Simulation Results

To verify the effectiveness of our ISSA protocol and beam scheduling algorithm, extensive simulations for performance evaluation are implemented in this section. We give the capacity of the ISSA protocol, and compare it with the SSA protocol. Then, the system throughput simulation that combines ISSA with beam scheduling is conducted, and we compare the throughput and collision probability with round robin in different traffic arrival rates. The throughput is defined as the number of packets recovered successfully at the satellite per beam-hopping slot/cycle and we assume that STs transmit only one packet per beam-hopping slot. The other simulation parameters are shown in Table 2.

**Figure 9:**
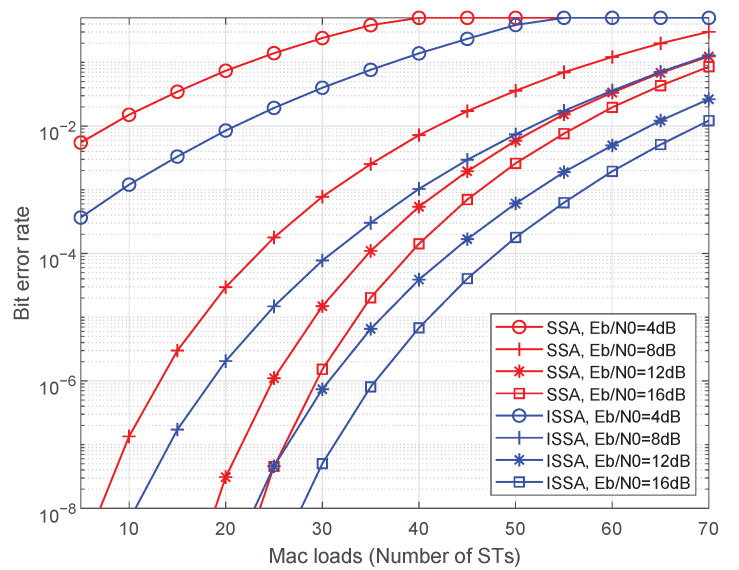
BER vs. number of STs in a cell.

Figure 9 depicts BER of our proposed ISSA and SSA protocols. The number of STs which send a packet to the satellite at a beam-hopping slot is 5∼70 in a cell and we assume that the received signals at the satellite from all STs keep the same Eb/N0, which is set as 4 dB, 8 dB, 12 dB, and 16 dB. From this figure, the capacity (the maximum mac loads or the maximum number of ST accesses to the satellite successfully using a beam at one beam-hopping slot) with different BERs can be achieved. It shows that as the BER requirement is $10^{-7}$, the beam can accommodate 18, 30 and 35 STs with Eb/N0 = 8 dB, 12 dB, 16 dB, respectively, using the ISSA protocol. In practice, the capacity may be higher for the above data when the discriminative equivalent isotropically radiated power (EIRP) brings diverse received power. The system can employ the diverse received power to apart information from the mixed signal easily with some approaches, such as SIC. However, SIC is so complicated for a satellite and cannot realize real-time decoding, and the performance improvement is only 2∼3 dB, which is not a cost-effective scheme. As shown in Figure 9, the capacity using the ISSA protocol is about 16.7% larger than the SSA protocol when BER is $10^{-6}$.

**Figure 10:**
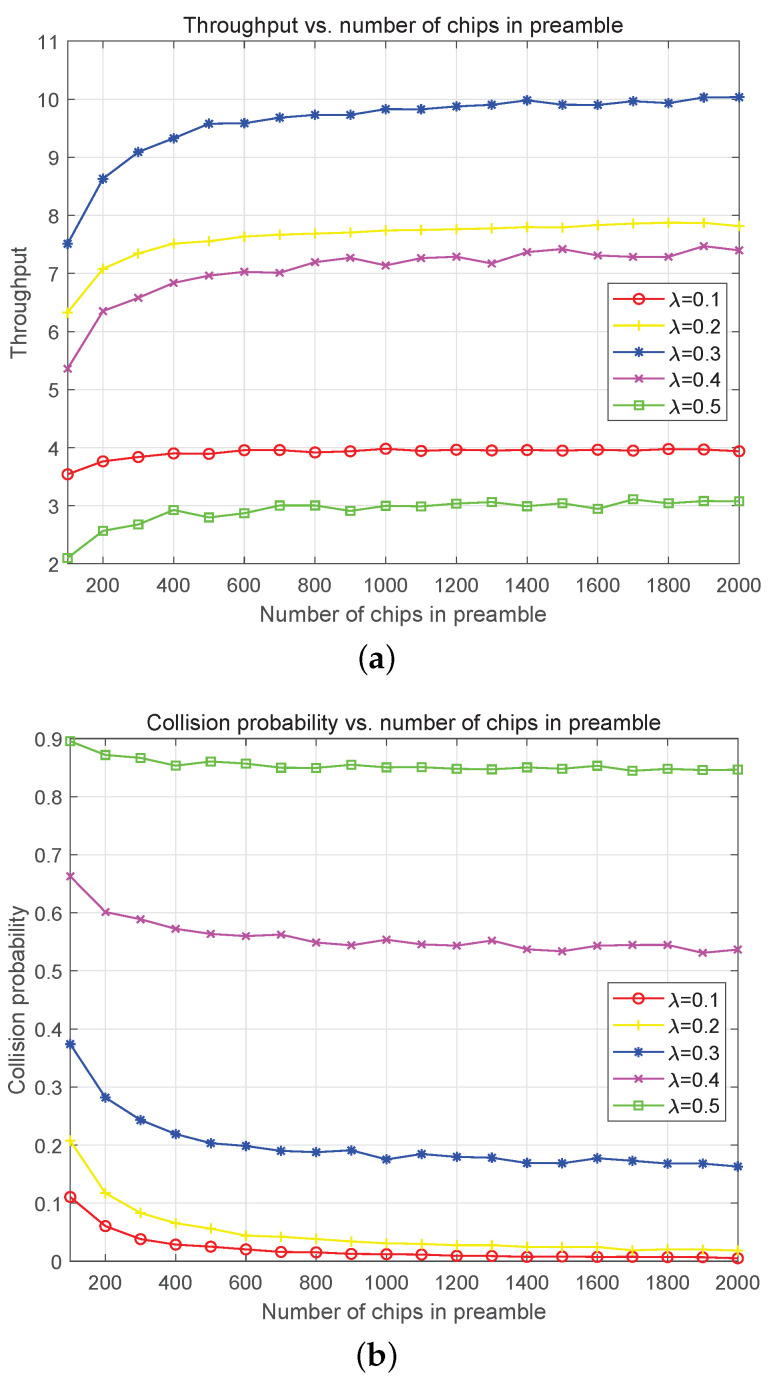
Throughput (packet per beam-hopping slot) and collision probability simulation with different number of chips in the preamble. (**a**) Throughput in one access vs. Number of chips in the preamble. The number of STs is 40 and Eb/N0 = 8 dB. (**b**) Collision probability in one access vs. Number of chips in the preamble. The number of STs is 40 and Eb/N0 = 8 dB.

The simulation of throughput (packets per beam-hopping slot) in one beam-hopping slot against the number of chips in the preamble with different Poisson arrival rates is presented in Figure 10a. The throughput obviously improves with the increase of chip number in the preamble for the optional chips, it is more and the collision probability is lower. The maximum throughput appears at λ=0.3 for the packet arrival and collision achieving a good trade-off. It can be seen from Figure 10b that the collision probability reduces remarkably at λ=0.3 compared with λ=0.5 and λ=0.4. In Figure 10b, the collision probability reduces with a decrease of λ and an increase of chip number in the preamble. When λ=0.3, the collision probability is reduced to a relatively low level.

**Figure 11:**
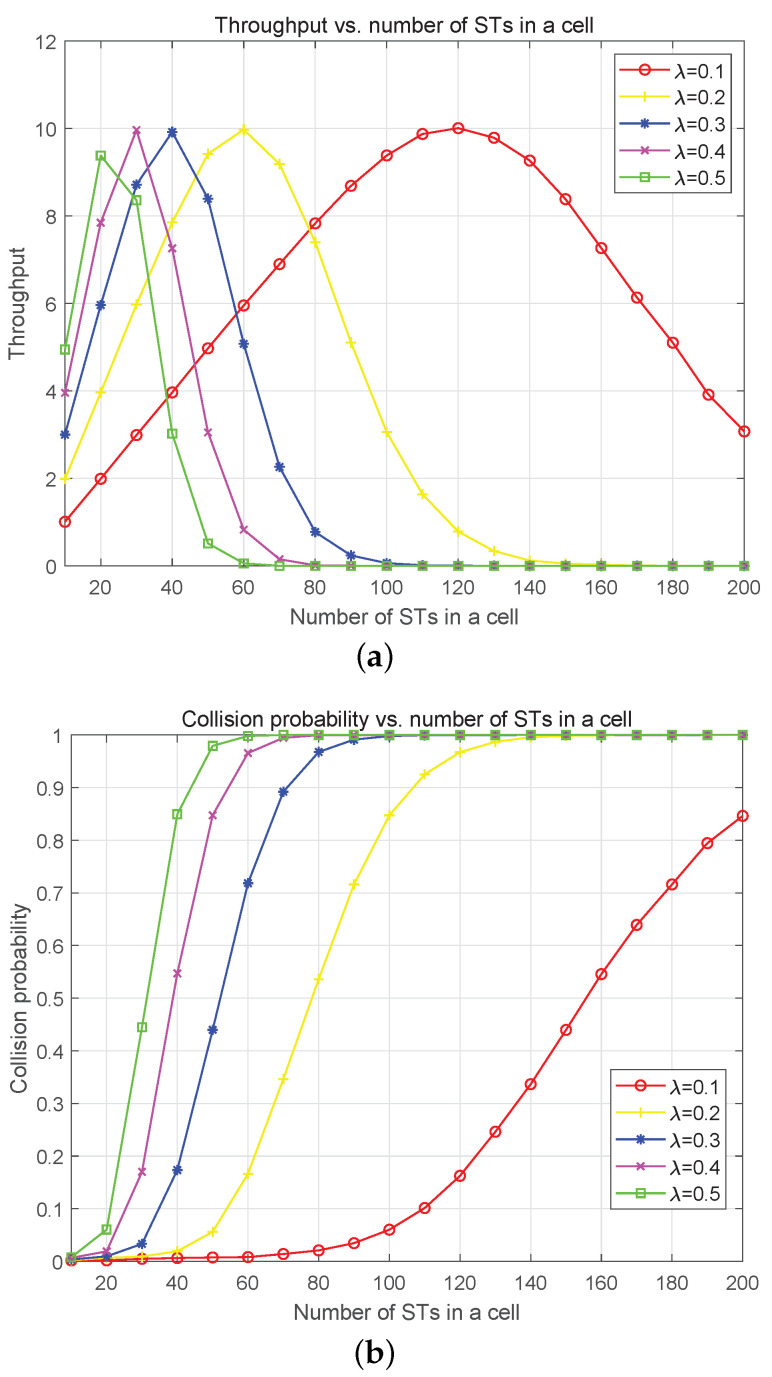
Throughput (packet per beam-hopping slot) and collision probability simulation with different number of STs in a cell. (**a**) Throughput in one access vs. Number of STs in a cell. The number of chips in the preamble is 2000 and Eb/N0 = 8 dB. (**b**) Collision probability in one access vs. Number of STs in a cell. The number of chips in the preamble is 2000 and Eb/N0 = 8 dB.

The throughput (packets per beam-hopping slot) and collision probability against the number of STs in a cell is simulated and presented in Figure 11. Figure 11a shows that maximum throughput exists with the change of ST number and the collision probability increases with increase of the ST number in a cell. The maximum throughput is about 10 packets/slot, which is associated with Eb/N0 and the maximum access capacity. At the same time, when the packet arrival rate exceeds 12 packets/slot, the system throughput decreases for the increasing collision probability. For the simulation of collision and the number of STs in the cell, the obvious result is that the collision probability increases with the increase of the number of STs in the cell and the average arrival rate of traffic.

**Figure 12:**
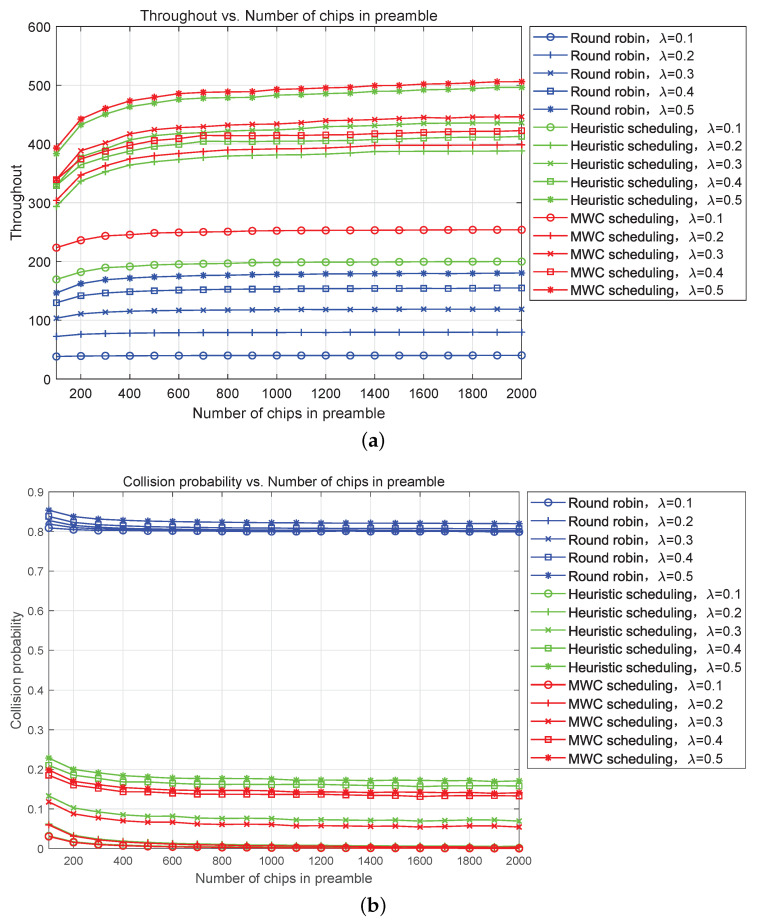
Throughput (packet per beam-hopping cycle) and collision probability simulation with different beam scheduling algorithms. (**a**) Throughput vs. Number of chips in the preamble. (**b**) Collision probability vs. Number of chips in the preamble.

Figure 12 gives the throughput (packets per beam-hopping cycle) and collision probability of our proposed heuristic and MWC beam scheduling algorithms in a beam-hopping cycle against the number of chips in the preamble. The setting access capacity is 30 and the distribution of STs in cells is [500, 300, 0, 3, 1, 3, 11, 13, 15, 27, 16, 24, 21, 25, 8, 13, 7, 8, 2, 1, 1, 0, 1, 0, 0]. Two cells cover almost 80% STs, and the performance of the scheme with beam scheduling is much better than round robin’s. The MWC scheduling algorithm assigns larger weight to user distribution to achieve the goal of maximizing throughput or access capacity in satellite IoTs. From this figure, we can see that the throughput is improved significantly with beam scheduling, especially in the condition of the number of chips in the preamble is over 500 and the performance of MWC scheduling is better than heuristic scheduling. The collision probability is reduced much and kept under 20% with beam scheduling. However, the collision probability of round robin is almost kept around 80%, which implies that many STs cannot access effectively. The collision probability of MWC scheduling is lower than heuristic scheduling, and the gap decreases gradually with the increase of the number of chips in the preamble. Further simulation indicates that the performance with beam scheduling in condition of nonuniform distribution of STs is approaching the performance of uniform distribution, which is an anticipated result.

**Figure 13:**
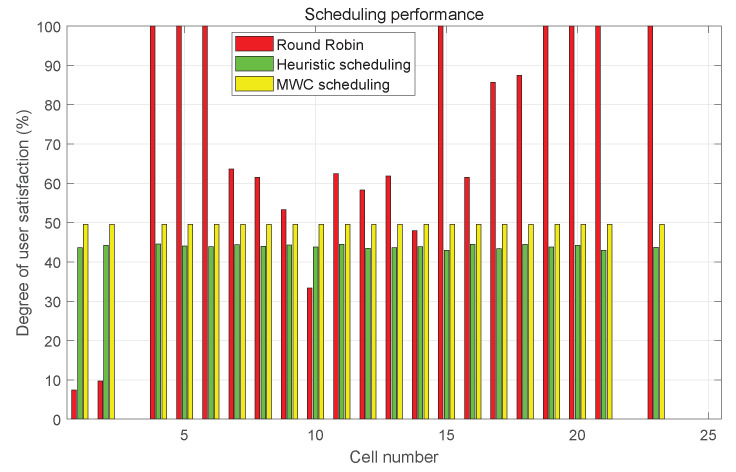
Degree of user satisfaction with different scheduling approaches.

Figure 13 presents the slot allocation with the same user distribution as Figure 12, and we set λ=0.3 and the number of chips in the preamble is 1000. The degree of user satisfaction is used to measure the scheduling with different algorithms. It can be seen from the figure that the degree of satisfaction is much lower in cells that hold many STs with round robin but 100% in cells with few STs. However, we prefer to be able to guarantee the service requirements of large-volume cells to fulfill more users and more important services. The heuristic and MWC algorithms can adjust the beam scheduling according to the ST distribution and service requirements to improve the degree of satisfaction for cells with large number of STs significantly. In addition, the performance of MWC algorithm is better than the heuristic algorithm for MWC being able to find the optimal combination of cells to maximize the system throughput. Of course, the MWC algorithm is also more complex than the heuristic algorithm, and more calculation time and resources are required.

## 6. Remarks

In the different scenarios, beam-hopping may face some unpredictable problems, which are discussed in the following.

- Doppler shift. In LEO constellation, the rapid movement of LEO satellite relative to the ground results in a large and changed Doppler shift, also includes the change of delay and propagation loss. This has a huge and unfavorable influence on the time and frequency synchronization between satellite and terrestrial users and the power control. To deal with this problem, the prediction of satellite position and velocity based on ephemeris and telemetered data will play an import and key role. The related scheme has been verified on-orbit in the Chinese Global Broadband Multimedia Beta satellite, whose orbital altitude is 950 km. The result indicate that our scheme works well and the Doppler shift has been reduced to 10∼200 Hz with Ka frequency.
- LEO satellite constellation. In our designed beam-hopping satellite system, the beam-hopping interval is at the millisecond level and the beam-hopping cycle is at the second level. While the visible time of LEO satellite is about minutes to tens of minutes, which is much longer than beam-hopping interval and beam-hopping cycle. Through the calculation of the azimuth and elevation, the largest changes in a beam-hopping interval are below 0.5° when the orbital altitude is 1000 km, which has little effect on the performance. The beam pointing does not need to be adjusted within a beam-hopping interval. In addition, the division of service areas ensure that the satellite can cover STs even at the edge of the service area in LEO constellation. In addition, the division of service areas has been discussed in our authorized patent. However, if the orbital altitude is 500 km or even lower or the designed beam-hopping interval is long, the change of azimuth and elevation will be large and bring greater difficulties to the system design.
- Geostationary surface (GeoSurf) constellation. GeoSurf constellation has been proposed in [27] to ease the system design. With GeoSurf constellation, steering antennas will not be needed, and the tropospheric propagation fading is minimized both for the stationary fading due to water vapor and oxygen, and for rain attenuation. While, the Doppler shift is largely minimized for the connected satellite and would always be viewed almost at the local zenith. Furthermore, the footprint of a single satellite is always the same, therefore making the design easier. The main problem is the large number of satellites crowding the polar region, resulting in serious system self-interference. However, it can be eased by changing the orbital inclination and turning off some satellite loads at high latitudes. In [28], the authors give the evaluation results on both the output of a direct channel and the interference coming from the orthogonal channel in a GeoSurf satellite constellation.

## 7. Conclusions

In this paper, we study the random access based on the spread spectrum technology in beam-hopping satellite systems. The ISSA protocol has been proposed, and the heuristic and maximum-weighted clique beam scheduling algorithms are used to allocate the slots for cells. These algorithms consider the ST distribution, interference avoidance, fairness and channel condition comprehensively. The simulation results show that the ISSA protocol achieves large throughput gain and the beam scheduling algorithms bring benefit compared with the round robin scheme, especially in the scenario of nonuniform distribution of STs.

## Figures and Tables

**Table 1:** Rain attenuation in different cities with 99.9% and 99.99% availability.

City	Rain Attenuation (dB)	
	99.9% Availability	99.99% Availability
Beijing	10.1458	24.6919
Shanghai	11.0639	26.9400
Sanya	11.9523	28.8812
Chongqing	11.1984	27.2695

**Table 2:** Simulation parameters.

Parameter Type	Value
frame length (bits)	128
encode style	Convolutional/Turbo
code rate	1/2
modulation style	QPSK
spread spectrum factor	128
spread spectrum code	m-sequences
Eb/N0 (dB)	4, 8, 12, 16
preamble length (chips)	100–2000
mean value of Poisson arrival	0.1, 0.2, 0.3, 0.4, 0.5
symbol rate (ksyms/s)	4.8
number of beams	4
number of cells	25
number of STs	1000

## Data Availability

Not applicable.

## Abbreviations and Notations

The following abbreviations and notations are used in this manuscript:

Abbreviations	
AWGN	Additional Gaussian White Noise
BER	Bit Error Rate
BH	Beam-Hopping
BQP	Binary Quadratic Programming
CDMA	Code Division Multiple Access
CRDSA	Contention Resolution Diversity Slotted Aloha
CSA	Coded Slotted Aloha
DLL	Double-Loop Learning
DSA	Diversity Slotted Aloha
ECEF	Earth-Centered Earth-Fixed
E-SSA	Enhanced-SSA
FBHCA	Flexible Beam-Hopping Control Algorithm
FEC	Forward Error Code
GA	Genetic Algorithm
GEO	Geostationary Earth Orbit
IRSA	Irregular Repetition Slotted Aloha
IoT	Internet of Things
ISSA	Improved Spread Spectrum Aloha
LEO	Low Earth Orbit
MADRL	Multi-Agents Deep Reinforcement Learning
maxUSWG	maximal User Service Weight Gain
MPMM	Multiplier Penalty and Majorization Minimization
MWC	Maximum Weighted Clique
NOMA	Non-Orthogonal Multiple Access
NRT	non-RT
PDF	Probability Density Function
QoS	Quality of Service
QPSK	Quadrature Phase Shift Keying
RA	Random Access
RT	Real-Time
SA	Slotted Aloha
SDP	Semi-Definition Programming
SIC	Successive Interference Cancellation
SINR	Signal to Interference plus Noise Ratio
SS	Spread Spectrum
SSA	Spread Spectrum Aloha
ST	Satellite Terminal
TDMA	Time Division Multiple Access
Notations	
E	Set of edges in auxiliary graph
G	Auxiliary graph
H	Set used to store the beam-hopping pattern
I	Set of slots
J	Set of beams
K	Set of cells
$N(v_{k})$	Set of all adjacent vertices for $v_{k}$
P	Set used to store the proportion of online STs
Q	Clique in the auxiliary graph
$Q_{m}$	Maximum weighted clique
Q	Clique set based on auxiliary graph
T	Set used to store the number of allocated slots to each cell
V	Cell set used to store the vertices in auxiliary graph
W	Weight set used to store the weights of all cells’ or vertices’
Θ	Beam angle set
*A*	Channel gain matrix
*B*	Beam bandwidth
$D_{k}$	Number of online STs in cell *k*
$G_{r}$	Receiving antenna gain matrix
$G_{t}$	Transmitting antenna gain matrix
$G_{T}\left(\theta \right)$	Gain of phased array antenna with off-axis angle θ
$H_{i}$	Backward channel gain matrix at slot *i*
*I*	Number of slots in a BH cycle
*J*	Number of beams
*K*	Number of cells
*M*	Number of chips in preamble
*N*	Number of phased array elements
$N_{0}$	Power of white Gaussian noise
$N_{pre}$	Number of chips in preamble
$P_{i}$	Transmitting power matrix at slot *i*
$R_{i,k}$	If cell *k* still needs service at slot *i*, $R_{i,k}=1$; Otherwise, $R_{i,k}=0$
*S*	Throughput expressed in packets/users per beam-hopping slot
*T*	Number of STs in a cell whose signal arrived at satellite in a BH cycle
$T_{max}$	Maximum throughput in a parameter setting
*X*	Beam occupancy condition
$g_{k,j}^{t}$	Transmitting antenna gain from cell *k* using beam *j*
$g_{j}^{r}$	Receiving antenna gain at satellite on beam *j*
$h_{k,j}$	Backward link gain from ST in cell *k* to on-board receiver using beam *j*
$p_{k,j}$	Total transmitting power in cell *k* using beam *j*
$p_{0}$	Packet recovery probability
$v_{k}$	Vertex in auxiliary graph used to represent cell *k*
$w_{k}$	Weight of cell *k* or vertex $v_{k}$
$x_{i,j,k}$	Illuminating state, if beam *j* illuminate cell *k* at slot *i*, $x_{i,j,k}=1$; Otherwise, $x_{i,j,k}=0$
$x_{k,j}$	The illuminating state of cell *k* using beam *j*
$\Gamma_{i,j,k}$	SNIR of receiving signal at on-board receiver from cell *k* using beam *j* at slot *i*
$\alpha_{j}$	Channel gain of beam *j* over backward link
γ	Receiving SNR before hybrided at satellite
$\gamma^{\prime }$	Receiving SNR after hybrided at satellite
η	Spectrum Spread factor
$\eta^{\prime }$	Antenna efficiency
θ	Off-axis angle of phased array antenna
$\theta_{c}$	Angle of interference avoidance threshold
$\theta_{k_{m},k_{n}}$	Angle between two beams illuminate cell $j_{m}$ and $j_{n}$
$\theta_{i,k_{m},k_{n}}$	Angle between two beams illuminate cell $j_{m}$ and $j_{n}$ at slot *i*
λ	Poisson arrival rate
τ	Beam-hopping cycle denoted as the number of slots
$\tau^{\prime }$	Minimum number of slots allocated to a cell in a BH cycle
